# Effect of Rice Husk Addition on the Hygrothermal, Mechanical, and Acoustic Properties of Lightened Adobe Bricks

**DOI:** 10.3390/ma18143364

**Published:** 2025-07-17

**Authors:** Grégoire Banaba, Sébastien Murer, Céline Rousse, Fabien Beaumont, Christophe Bliard, Éric Chatelet, Guillaume Polidori

**Affiliations:** 1 Interdisciplinary Research on Society-Technology-Environment Interactions (InSyTE), Université de Technologie de Troyes, F-10004 Troyes, France; gregoire.banaba@utt.fr (G.B.); eric.chatelet@utt.fr (É.C.); 2 Institut de Thermique, Mécanique, Matériaux (ITheMM), Université de Reims Champagne-Ardenne, F-51100 Reims, France; sebastien.murer@univ-reims.fr (S.M.); celine.rousse@univ-reims.fr (C.R.); fabien.beaumont@univ-reims.fr (F.B.); 3 UMR 7312 CNRS, Institut de Chimie Moléculaire de Reims (ICMR), Université de Reims Champagne-Ardenne, F-51100 Reims, France; christophe.bliard@univ-reims.fr

**Keywords:** adobe, lightened earth, rice husk, moisture buffer value, compressive strength, thermal performance, acoustics

## Abstract

In the context of efforts to reduce greenhouse gas emissions in the building sector, the reintegration of traditional earthen construction into modern architectural and renovation practices offers a sustainable alternative. To address the mechanical and water-resistance limitations of adobe bricks, the use of agricultural waste—such as rice husk—is increasingly being explored. This experimental study evaluates the effects of rice husk addition on the mechanical, hygrothermal, and acoustic properties of adobe bricks. Two soil types—one siliceous and one calcareous—were combined with 1, 2, and 3 wt% rice husk to produce bio-based earthen bricks. The influence of rice husk was found to depend strongly on the soils’ mineralogical and granulometric characteristics. The most significant improvements were in hygrothermal performance: at 3 wt%, thermal conductivity was reduced by up to 35% for calcareous soil and 20% for siliceous soil, indicating enhanced insulation. Specific heat capacity also increased with husk content, suggesting better thermal inertia. The moisture buffering capacity, already high in raw soils, is further improved due to increased surface porosity. Mechanically, rice husk incorporation had mixed effects: a modest increase in compressive strength was observed in siliceous soil at 1 wt%, while calcareous soil showed slight improvement at 3 wt%. Acoustic performance remained low across all samples, with minimal gains attributed to limited macro-porosity. These findings highlight the importance of soil composition in optimizing rice husk dosage and suggest promising potential for rice husk-stabilized adobe bricks, especially in thermally demanding environments.

## 1. Introduction

Earth is undoubtedly one of the oldest building materials in human history. Persian, Assyrian, Egyptian, and Babylonian civilizations made abundant use of it [1]. It is estimated that 8–10% of the world’s population lives in earthen constructions, going up to 20–25% in developing countries [2]. In Europe, although largely forgotten today, earth constructions still form part of the daily landscape [1]. In France, one or more traditional earth construction techniques are found in all regions, except mountainous areas, in both rural and urban centers.

Today, the construction sector accounts for 33% of greenhouse gas emissions, 40% of material consumption, and 40% of global waste production [3]. With increasing awareness of resource depletion and the consequences of climate change over the past two decades, earth construction techniques have resurfaced as efficient and ecological solutions to reduce energy consumption and greenhouse gas emissions in the construction and housing sectors. However, despite its excellent carbon footprint and hygrothermal properties, raw earth remains less attractive due to its high sensitivity to water and low compressive strength compared to widespread cement-based materials. To palliate this, stabilization of earth with mineral binders such as cement or lime is often performed [4]. Unfortunately, even in small quantities, these stabilizers drastically affect the environmental cost and prevent the recyclability of these materials [5]. Consequently, recent studies suggest that using organic binders from plant fibers or agricultural waste as organic reinforcement could offer a more sustainable alternative to mineral stabilizers [6,7,8,9] due to favorable physico-mechanical properties [10,11,12].

Rice husk, the protective layer surrounding the rice grain during growth, consists mainly of silica and organic biopolymers and represents about 20% of the rice grain’s mass after harvest. It is a byproduct of rice milling, produced during the dehulling process.

Considering the potential benefits of plant waste in improving raw earth brick properties, this article focuses on the use of rice husk to enhance the hygrothermal, mechanical, and acoustic properties of adobe bricks, handmade or molded in their plastic state and air-dried [13].

Research on using raw, non-calcined rice husk in adobe manufacturing remains limited. Ouedraogo et al. [14] demonstrated that adding rice husk to a low-plasticity clay (plasticity index = 14%) enhanced compressive strength and reduced thermal conductivity. The best compressive strength (3.6 MPa) was achieved with 0.4% rice husk addition. Samson et al. [15] evaluated the mechanical and physical properties of adobes with 0–2.5% rice husk by weight, noting improvements in mechanical strength and reductions in shrinkage and water absorption. Ige and Danso [16] showed that stabilizing adobes with 0.75% rice husk and 10% lime improved compressive and tensile strength by 62% and 95%, respectively. Antunes et al. [17] explored biosourced composite panels with rice husk, finding a 50% reduction in thermal conductivity and a 20% increase in humidity buffering. Buratti et al. [18] studied the thermal and acoustic performance of rice husk panels, finding thermal conductivity comparable to traditional insulation and strong sound absorption coefficients. Vatani Oskouei et al. [19] showed lower compressive and tensile strength for rice husk composites compared to palm fiber ones. Akinyele et al. [20] found that a 2% addition of 300 μm sized rice husk particles gave the highest compressive strength (5.47 MPa) in fired clay bricks. Sutas et al. [21] compared rice husk and rice husk ash additives and found that 1% husk addition provided the best compressive strength (4 MPa), while 2% ash addition further improved properties. Bazuhair [22] added up to 3 wt% rice husk to compressed earth blocks and found a decrease in shrinkage and drying time, while compressive strength improved by 25% between 1 and 3 wt%. Sasui et al. [23] compared adobes containing 2 wt% of straw and 2 wt% of rice husk and showed that the compressive strength of adobes with rice husk content was significantly higher. By incorporating up to 9 wt% of rice husk in clayey soil adobes, Lertwattanaruk and Tungsirisakul [24] stated that the mechanical resistance is optimal at 3 wt% content, while reducing drying shrinkage.

The aim of the present study is to optimize the hygrothermal, mechanical, and acoustic properties of rice husk-stabilized adobes, considering the elemental composition of the earth used. The goal is to analyze formulations offering optimal mechanical strength, thermal insulation, moisture regulation, and acoustic performance, contributing to sustainable, environmentally friendly construction capable of supporting at least one story. Two soil samples from different localities will be mixed with 1, 2, and 3 wt% rice husk, and physical, mechanical, thermal, moisture, and acoustic tests will be conducted to guide the construction sector in using rice husk as an eco-friendly stabilizer for adobe production.

## 2. Materials and Methods

### 2.1. Geographic Origin of the Studied Soils

**Figure 1:**
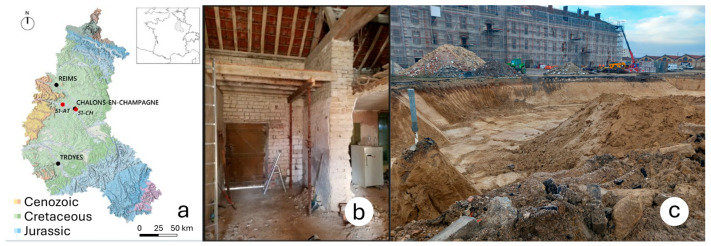
(**a**) Geographic origin of the soil samples—from Polidori et al., 2025 [25]; (**b**) 51-AT adobes collected from a traditional barn; (**c**) excavation site of 51-CH soil.

Two soil samples were collected from Athis and Châlons-en-Champagne, two localities in the Champagne-Ardenne region of France (Figure 1a). These two soil samples are hereafter referred to as 51-AT and 51-CH, respectively: the two digits correspond to the number of the French department, and the two letters correspond to the first two letters of the town or village from which the soils originate. Champagne-Ardenne is situated in northeastern France and constitutes a transitional region to the east of the Paris Basin. Stretching 300 km from north to south and 200 km from east to west, it covers an area of 25,600 km^2^. The region is composed of Cenozoic plateaus in the west, Jurassic and Cretaceous plateaus in the east, and Quaternary alluvial valleys. To the north, it is bordered by the remnants of the ancient Paleozoic Ardenne mountain range. As a result, it presents a wide diversity of soil types. The Chalky Champagne, a vast chalk plain, occupies the central part of the region [25].

### 2.2. Origin of the Soil Used

The soil used in this study, denoted 51-AT, originates from adobes collected in the load-bearing walls of a demolished traditional barn. Their dimensions are found to be $296.3\times 141\times 101\;{mm}^{3}$ on average (Figure 1b, Polidori et al., 2025 [25]). Châlons soil (51-CH) is a site excavation soil (Figure 1c), collected at a depth of around 4 m below the natural soil surface. It is expected that variations in the physical, chemical, and mineralogical properties of raw earth, resulting from its specific geological and geographical origin, influence its functional performance across mechanical, thermal, hydric, and acoustic domains.

### 2.3. Overall Characteristics of Rice Husk

**Figure 2:**
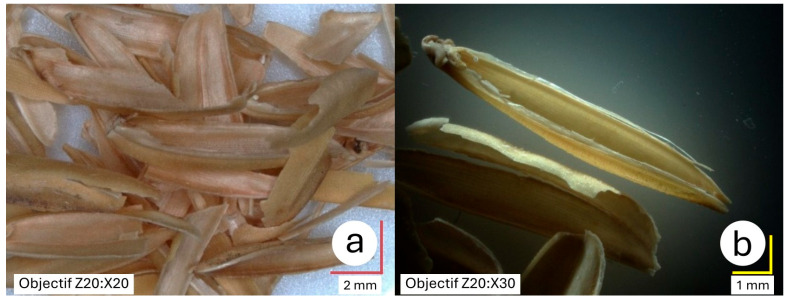
Bulk rice husk (**a**); rice husk in detail (**b**).

The rice husk used in this study originates from the Camargue region, the heartland of French rice cultivation, which spans an area of 146,000 hectares. The rice harvesting process begins with separating the grains from the straw in the harvested sheaves, followed by the removal of impurities such as insects, minerals, and plant debris. The grains are then subjected to a drying process before undergoing dehusking. This final step separates the grain from its husks, which appear as half-shells (see Figure 2).

Rice husk accounts for approximately 20% by mass of the whole unhulled grain. In France, around 15,000 tons of rice husks are produced annually in the Camargue region [26]. Rice husk is composed of 70–80% organic matter (including 38% cellulose, 22% lignin, 18% pentosan, and 2% other organic substances) [27], which is lower than most other lignocellulosic resources. The remaining 20% is primarily amorphous silica, concentrated mainly on the outer surface of the husk [28]. Rice husk is virtually rot-resistant and impervious to insect attacks.

The management of this agricultural waste includes its reuse as a fertilizer for soil enrichment, as a fuel for electricity or heat production, or as construction material in the building sector when calcined [29].

### 2.4. Granulometry of Soil Samples

The particle size analysis was conducted in accordance with ISO 11277:2020 [30], using wet sieving with nine sieves of decreasing mesh size ranging from 10 mm to 25 μm on randomly selected samples from each soil type.

To access particle size fractions smaller than 25 μm, laser diffraction was employed using a Malvern Mastersizer 2000 granulometer (Malvern Instruments Ltd., Worcestershire, UK). The consistency between the two granulometric techniques—one based on mass and the other on volume—is ensured by verifying that the absolute densities of the corresponding samples are comparable [25].

### 2.5. Morphology and Elemental Composition of Soil Samples and Rice Husk

The morphology and elemental chemical composition of the soil and rice husk samples were determined using a Hitachi TM3030Plus tabletop scanning electron microscope (SEM) coupled with a Swift ES3000 energy-dispersive X-ray spectroscopy (EDXS) system (Hitachi High-Tech Corp, Tokyo, Japan). X-ray spectra were acquired at an acceleration voltage of 15 kV with an acquisition time of 300 s.

For scanning electron microscopy observations, the specimens were coated with a conductive Au-Pd layer via sputter coating to prevent charging effects (i.e., electron accumulation on the surface of the material), which can compromise image quality. Non-conductive samples are unable to dissipate electrical charges, resulting in image instability and inaccurate EDXS analysis. Therefore, the soil and rice husk samples were crushed and compressed into pellets for EDXS analysis. Each experiment was repeated at least three times to ensure reproducibility of the results.

To determine the size distribution of the rice husks, a set of approximately one hundred randomly selected husk samples was measured using a Keyence VHX-970F digital microscope (Keyence Corporation, Osaka, Japan). Following these measurements, a scatter plot was generated, allowing for the calculation of the average length and width of the rice husks.

### 2.6. Measurements of CaCO_3_ and Water Contents

Decarbonization was performed using a custom-built single-unit Scheibler apparatus in accordance with the NF EN ISO 10693:1995 standard [31]. An estimation of the carbonate content in the soil samples was thus provided [25]. Moisture content was determined using a Sartorius MA100 high-precision moisture analyzer (Sartorius AG, Göttingen, Germany).

### 2.7. Adobe Manufacturing and Drying

**Figure 3:**
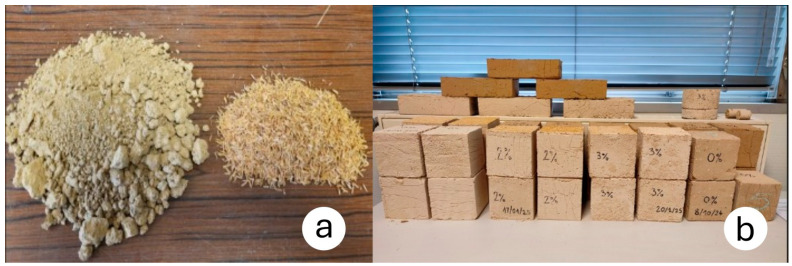
Overview of the soil and rice husk volumes for a 3 wt% content (**a**); drying of cubic earth-rice husk samples (**b**).

For mechanical, hygrothermal and acoustic tests, cubic and cylindrical samples are made from each of the two soils, with rice husk contents of 1, 2, and 3 wt% (Figure 3a), and then air-dried for 28 days in a controlled environment (22 °C, 55% RH), as seen in Figure 3b.

### 2.8. Compression Tests

Given that earthen architectural design is predominantly governed by compressive stresses, compression tests were conducted using a Zwick Roell Z050 testing machine (ZwickRoell, Ulm, Germany) equipped with a 50 kN load cell. The compression rate was set at 8 mm/min in accordance with the specifications of standard NF XP P13-901 [32], resulting in specimen failure within 1 to 2 min.

**Figure 4:**
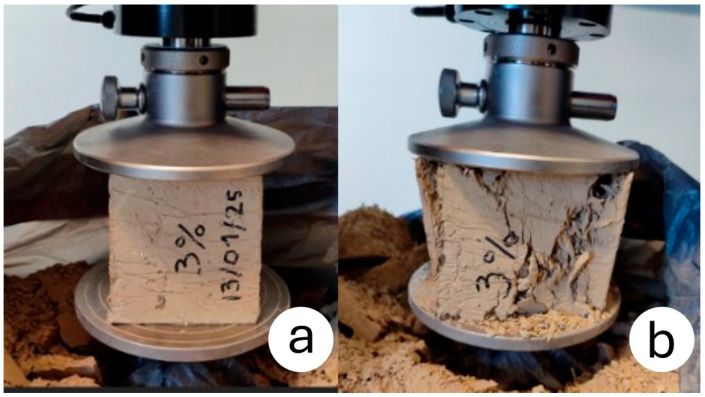
Compression test on a 3 wt% rice husk sample. (**a**) Specimen in place between compression platens; (**b**) Crushed specimen with corresponding failure mode.

In this study, four adobe bricks with cubic dimensions of $100\times 100\times 100\;{mm}^{3}$ made from each soil type, stabilized with 1%, 2%, and 3% rice husk content, were subjected to compression tests, as seen in Figure 4. To ensure optimal contact between the sample surfaces and the compression platens, the faces were manually smoothed using sandpaper.

### 2.9. Thermal Tests

The thermal properties evaluated for both the adobe samples and the rice husk were thermal conductivity, specific heat capacity, and thermal diffusivity. The measurements were carried out using an ISOMET 2114 thermal properties analyzer (Applied Precision, Ltd., Bratislava, Slovakia). For the adobe samples, measurements were conducted based on heat flux pulses applied to the surface of the specimens. For the rice husk, a cylindrical container with dimensions ϕ16×32 cm was filled and compacted to a mass of 1 kg under controlled conditions of 20.9 °C and 54% relative humidity (RH). The related thermal characteristics were determined using an API 210412 contact probe (Applied Precision, Ltd., Bratislava, Slovakia).

### 2.10. Moisture Buffer Value (MBV)

The moisture buffering value (MBV) assesses a material’s ability to regulate fluctuations in relative humidity within an enclosed space, thereby providing insight into the hygrometric comfort experienced by building occupants [25]. The MBV measurement follows the protocol described in the Nordtest project [33], which classifies moisture buffering performance from negligible to excellent. To ensure measurement reproducibility, four parallelepiped adobe samples were prepared for each soil type. Each sample measured $10\times 10\times 4\;{cm}^{3}$ and was sealed on the lateral and bottom surfaces using waterproof adhesive tape.

Samples were conditioned at 23 °C and 50% relative humidity for 14 days and then subjected to daily RH cycles: 8 h at 75% RH followed by 16 h at 33% RH in a climatic chamber (Binder MKF 720, Tuttlingen, Germany). These cycles continued until the difference in mass variations measured over the last three cycles was less than 5%. The MBV value was then determined using the following equation:

(1) $$MBV=\frac{∆m}{A({RH}_{sup}-{RH}_{inf})}$$

where MBV denotes the moisture buffering value expressed in g/(m^2^·%RH), ∆m represents the mass variation during absorption and desorption, A denotes the surface area of the sample exposed to air, and ${RH}_{sup}$ and ${RH}_{inf}$ represent the upper (75%) and lower (33%) relative humidity levels, respectively.

### 2.11. Acoustic Tests

The sound absorption coefficient of the various samples was calculated over a frequency range of 125 to 4000 Hz using the impedance tube method (also known as Kundt’s tube), in accordance with ISO 10534-2 [34]. To this end, 30 mm diameter samples were used for high-frequency tests (f > 2000 Hz), while 100 mm diameter samples were used for low-frequency tests (f ≤ 2000 Hz). Since material thickness influences acoustic properties, cylindrical samples with thicknesses of 30 mm and 50 mm were prepared.

## 3. Results and Discussion

### 3.1. Particle Size Distribution of Soil and Rice Husk

**Figure 5:**
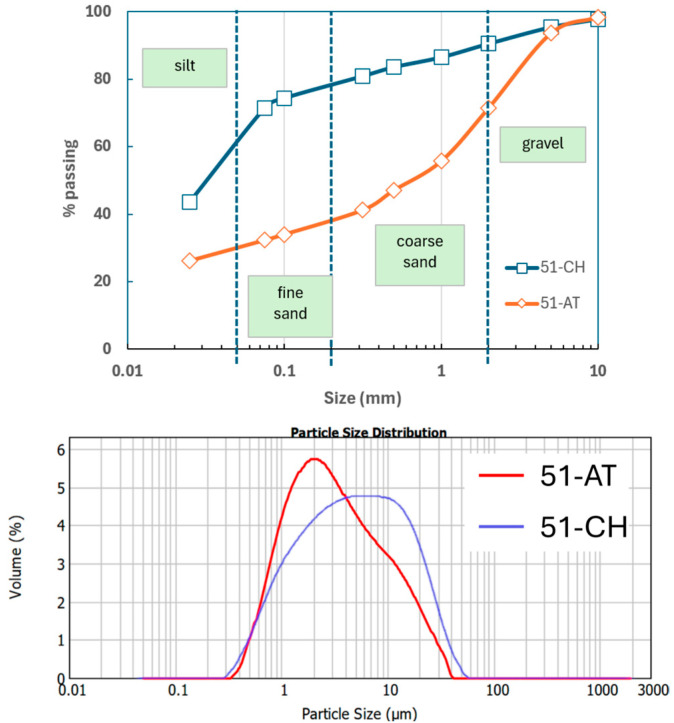
PSD of soil samples with wet sieving (**top**) and laser granulometry (**bottom**).

The particle size distribution (PSD) curves related to the two studied soils based on wet sieving and laser granulometry are presented in Figure 5.

Typically, PSD curves in Figure 5 (top) exhibit either a bell-shaped or a sigmoidal distribution pattern in the range 25 µm–10 mm, depending on the composition of the soils. These distinctive shapes have already been observed in a previous study [25] and are associated with the geological nature of the Champagne region (France). Gravels and stones (>2 mm), along with coarse sands (between 0.2 and 2 mm), are predominantly present in the limestone soil of 51-AT, while minimal to negligible gravel content is observed in the 51-CH soil. Additionally, the residual fraction, below 25 µm, serves as a support for laser granulometry, which is widely regarded as an effective technique for estimating clay content, typically corresponding to particle sizes below 2 μm or 5 μm in common soils, depending on the classification used [5,35]. Clay content appears to be a key parameter in earth building, since clay acts as a material binder and strongly affects the mechanical properties of earth-based construction. In the present study, focused on chalky soils, laser granulometry provides an upper limit for clay content, defined as particles below 2 μm. The associated curves are depicted in Figure 5 (bottom).

The calculation of clay content is based on the assumption that the relative mass of fine particles (≤25 µm) determined by sieve analysis is equivalent to the volume fraction (%) of particles ≤ 2 µm determined by laser granulometry. This assumption is only valid if the absolute density of the soil sample is equal to that of the fine particles ≤ 25 µm. We consider this condition to be satisfied, as the differences in density are 8.54% and 0.04% for 51-AT and 51-CH, respectively. This enables the estimation of clay contents at 10.27% for 51-AT and 11.30% for 51-CH. The literature suggests that clay content in the 5–29% range is generally considered acceptable [36,37], to strike a balance between sufficient compressive strength and moderate shrinkage, which may cause cracking.

**Figure 6:**
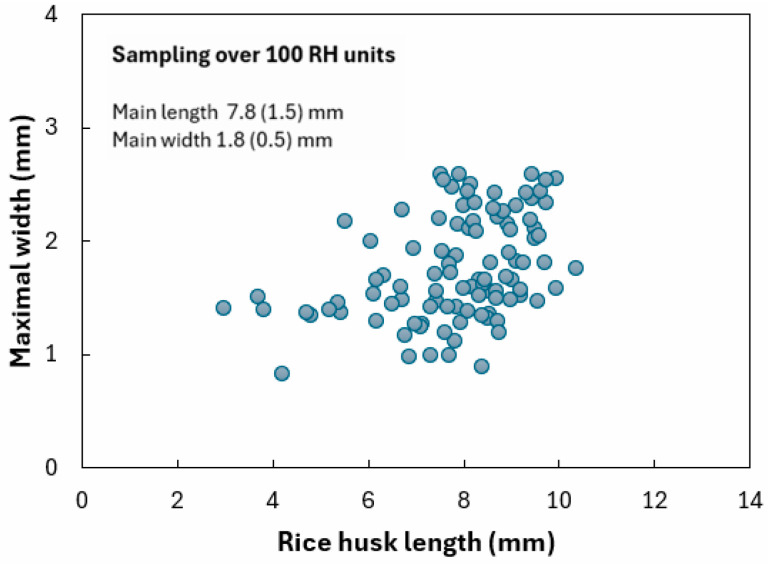
Size distribution of rice husk.

Concerning rice husk as an agrowaste used to lighten the adobe bricks, Figure 6 presents a scatter plot of the rice husk average dimensions, obtained using digital microscopy over a sampling of about 100 units. A relatively homogeneous distribution in size is observed, since the mean maximum length and width are found to be 7.8 (1.5) mm and 1.8 (0.5) mm, respectively. As such, rice husk can reasonably be considered a short vegetal component.

For comparison purposes, Chabannes et al. [29] report rice husk widths between 1 and 4 mm and maximum lengths around 10 mm; the orders of magnitude are comparable with our results.

### 3.2. SEM Morphology Analysis of Soil and Rice Husk

**Figure 7:**
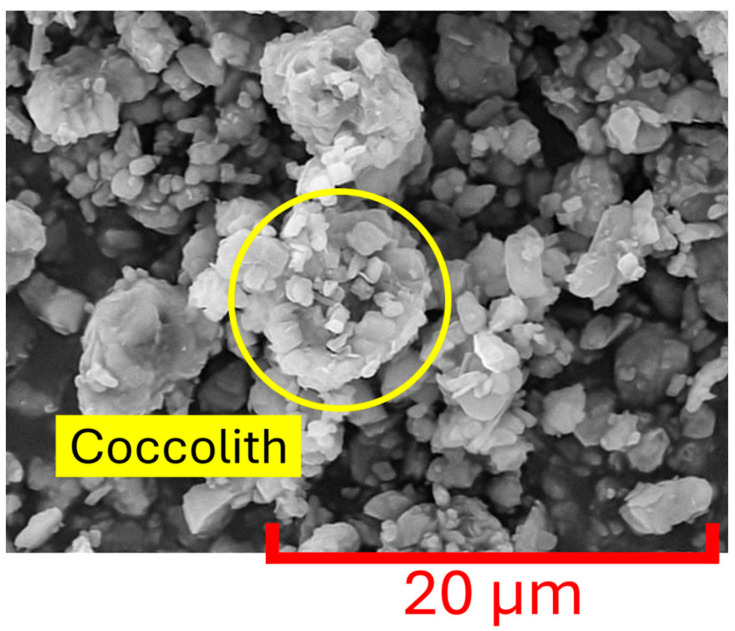
SEM image of 51-AT soil (×5000 magnification).

**Figure 8:**
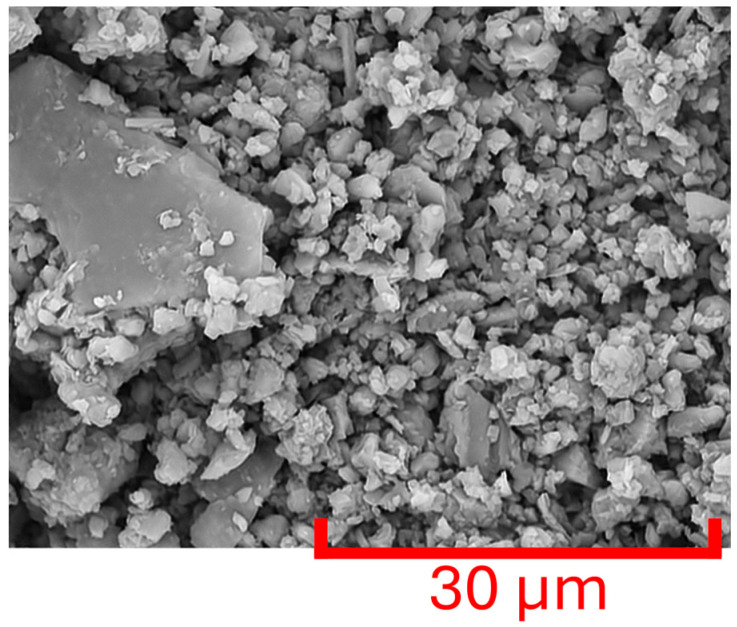
SEM image of 51-CH soil (×3000 magnification).

Figure 7 and Figure 8 present the microstructural characteristics of the two soil samples studied, obtained by scanning electron microscopy (SEM) at ×5000 and ×3000 magnifications, respectively.

In the 51-AT sample (Figure 7), a large amount of calcite micrograins is found, which is typical of limestone soils and subsoils. Coccoliths, calcitic particles produced inside the cells of unicellular marine algae known as *Coccolithophycea*, can also be observed, both whole and fragmented. These observations seem to be typical of chalk-based earth adobes, as already reported in a previous study [35], based on SEM analysis and confirmed by a decarbonization process at low granulometry. Moreover, XRD patterns could have further supported this result. Regarding the 51-CH sample (Figure 8), it appears that most of the clay content is constituted by crystalline calcite below 2 µm in size, as already reported for limestone soils [38]. More precisely, this strongly carbonated geomaterial exhibits porous aggregates of calcite nano- and micrograins, where elements are in point contact or create aggregates separated by larger pores, mainly in the form of cavities.

**Figure 9:**
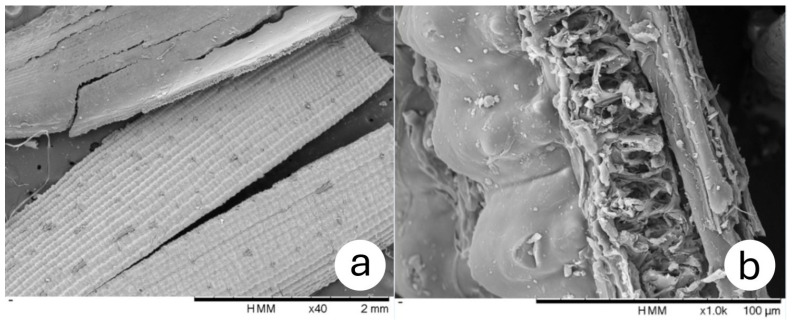
(**a**) Rice husk (×40 magnification); (**b**) cross-section (×1000 magnification).

For a closer look at the particularities of the rice husk structure, a view of the inner and outer faces is shown in Figure 9a and a cross-section in Figure 9b.

Scanning electron microscopy reveals peculiarities on the outer surface of the rice husk compared to other vegetal aggregates and shows that the epidermal cells of rice husk are arranged in the shape of ridges and linear furrows, punctuated by prominent conical protuberances. Park et al. [39], as well as Do Prado and Spinacé [40], also reported these irregularities on the outer surface of the rice husk. The epidermis of the inner concave surface is smoother than that of the outer surface [41]. The cross-section view (Figure 9b) also indicates a honeycomb structure between the inner and outer surfaces. The presence of this air-filled layer can be assumed to result in promising thermal properties.

### 3.3. Elemental Chemical Composition of Soil and Rice Husk Samples

**Figure 10:**
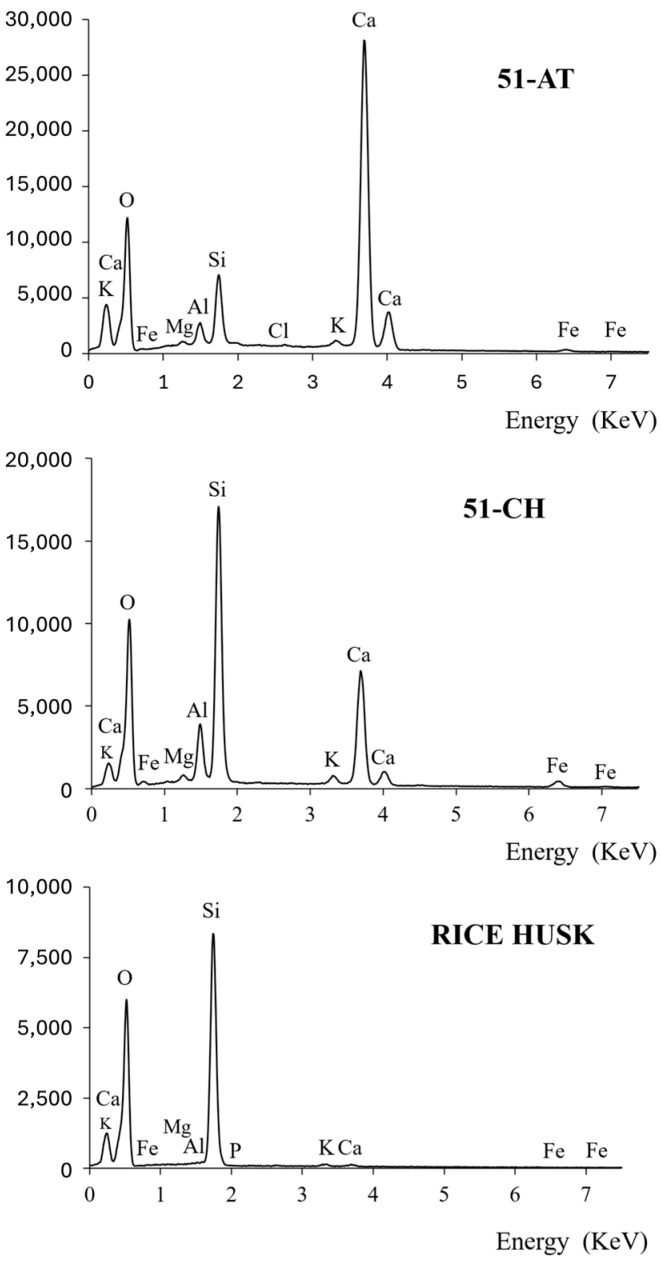
EDX spectra of 51-AT, 51-CH, and rice husk.

To confirm the trends observed by SEM in soils and rice husk, the elemental chemical composition of all samples was obtained by energy-dispersive X-ray spectroscopy (EDXS). Elemental spectra are presented in Figure 10.

The analysis of EDX spectra provides a qualitative overview of the presence of elements in the different samples. For instance, calcium content is found to be higher in the 51-AT soil, compared to 51-CH. The metallic elements Fe and Al, which are present in both soils (greater content in 51-CH), are only present in trace amounts in the rice husk. Furthermore, although silicon is found in both soils, it clearly appears as the main mineral element observed in rice husk.

Recent studies show that metallic elements play a dominant role in the mechanical cohesion of soils. Indeed, a strong positive linear correlation (Pearson’s coefficient 0.8) was observed between the peak compressive stress of soil samples and the cumulative atomic percentage of metallic elements [25] (and, by extension, the metallic oxides). This phenomenon can be attributed to the propensity of metallic elements to aggregate and bond with individual soil particles within the matrix, thereby augmenting soil cohesiveness [42].

To provide quantitative information, Table 1 summarizes the elemental composition, expressed in Atomic %, deliberately limited to the main elements found in soils and rice husk samples.

Results in Atomic % confirm that calcium, Ca, is the predominant mineral element in the 51-AT sample, with 21.5%. Furthermore, silicon Si is the most abundant mineral element in the 51-CH sample, associated with a significant proportion of aluminum Al and iron Fe. Consequently, the presence of metallic oxides, mainly SiO_2_ and Al_2_O_3_, which are the main constituents of clays, playing the role of cohesive binders in the manufacture of adobes, can reasonably be expected. It is found that rice husk (in its raw, unburnt form) contains approximately 24% silicon, in line with values reported in the literature [43].

The silicon-to-calcium ratio confirms the two soil families identified in the granulometric and morphological analyses, depending on whether this ratio is smaller than 1 (limestone soil such as 51-AT) or greater (siliceous soil such as 51-CH).

### 3.4. Physico-Chemical Characteristics of Rice Husk and Soil Samples

Soil specimens with varying rice husk contents were subjected to laboratory tests to assess their main physico-chemical characteristics, namely, absolute density, dry density, porosity, moisture content, pH, % CaCO_3_, % organic matter, and moisture buffer value. Table 2 summarizes the results for 51-AT and 51-CH soils with rice husk contents ranging from 0 to 3 wt%, along with those of rice husk alone.

The dry density of rice husk is found at 137.6 kg/m^3^, in line with values reported in the literature [44]. Regarding soil samples, the average dry density values at 0 wt% rice husk content are 1814.73 kg/m^3^ for 51-AT and 1736.87 kg/m^3^ for 51-CH. These values fall within the dry density range required for adobe masonry, which, according to certain authors, should vary between 14.13 and 25.07 kN/m^3^ [45] or between 1400 and 2200 kg/m^3^ [46].

**Figure 11:**
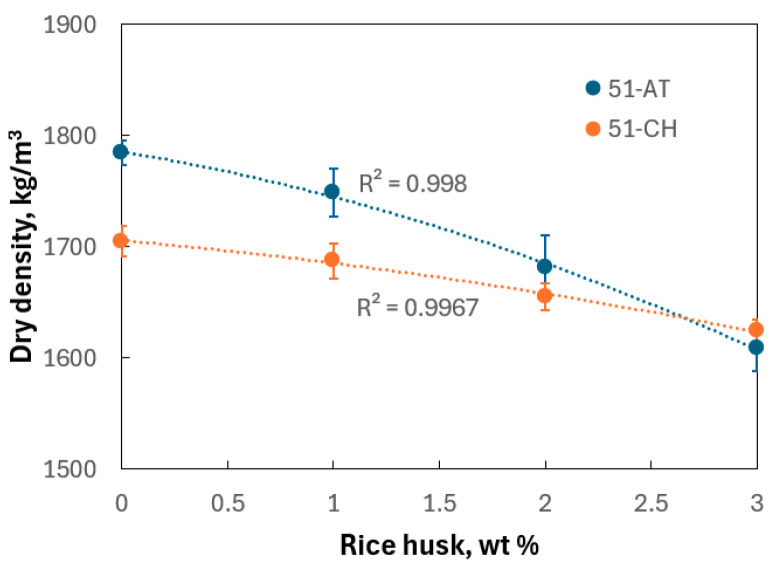
Dry density vs. rice husk content.

The curve in Figure 11 further indicates that the dry density of adobes decreases with increasing rice husk content, with a more pronounced effect observed for 51-AT (10.8%) than for 51-CH (4.9%). This trend is consistent with the incorporation of a plant-based additive whose density is approximately 11 to 12 times lower. The variation in dry densities of the adobes falls within the range reported for earth bricks modified with plant fibers and aggregates [47]. Similar observations were made by Babé et al. [48], who reported a decrease in density and an increase in porosity for earth bricks amended with peanut shells.

The MBV values obtained for 51-AT and 51-CH indicate that, even without the addition of rice husk, the soils can already be classified as very good to excellent moisture regulators. Since the incorporation of natural fibers increases the surface porosity of the composite—and consequently enhances its moisture buffering capacity—this classification can only improve [49,50]. To optimize time, it was deemed unnecessary to conduct MBV tests with rice husk addition.

From a chemical perspective, both soils exhibit near-neutral pH values (pH ≅ 7.8), with a slight calcareous tendency [25]. The soluble organic matter content was determined by evaporating the supernatant obtained after wet sieving and centrifugation, yielding values of 1.50% for 51-AT and 0.24% for 51-CH.

The measured calcium carbonate contents are consistent with the geological and pedological characteristics of the Champagne-Ardenne region from which the soil samples were sourced [25]. The carbonate content varies significantly, reaching 83.62% CaCO_3_ for 51-AT and 41.60% for 51-CH. In a recent study, Polidori et al. [51] demonstrated that even a high CaCO_3_ content of 71% in adobes can meet the structural requirements for earthen construction.

### 3.5. Compressive Test

**Figure 12:**
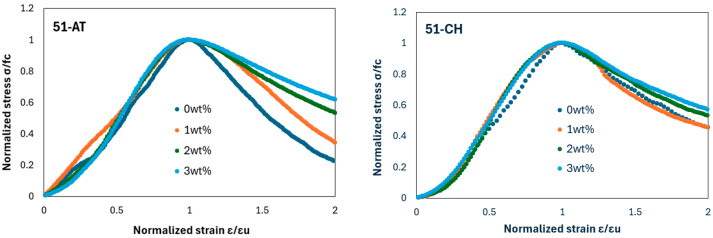
Normalized stress–strain curves for 51-AT (**left**) and 51-CH (**right**).

Mechanical tests were carried out on cubic adobe specimens produced by mixing each type of soil with rice husk content at 1 wt%, 2 wt%, and 3 wt%. Figure 12 presents the dimensionless compressive stress, a key parameter influencing material selection in earthen construction, denoted as $\sigma /f_{c}$, where $f_{c}$ is the peak compressive stress, plotted against the dimensionless strain $\varepsilon /\varepsilon_{u}$, where $\varepsilon_{u}$ corresponds to the strain at peak stress $f_{c}$.

Figure 12 shows that, overall, all compression curves exhibit a similar general behavior, characterized by an initial linear elastic branch followed by a nonlinear hardening phase leading up to the peak load, after which a ductile response is observed until failure. However, a significant variation in material behavior is noted for $\varepsilon /\varepsilon_{u}>1$ (i.e., beyond the peak stress point). This observation is consistent with findings reported by Illampas et al. [52]. The incorporation of rice husk appears to affect the ductile behavior of adobes. Although 51-AT and 51-CH raw soils display different behaviors in this regard, the addition of rice husk tends to lessen this difference. At 3% wt%, the normalized stress–strain curves are almost identical.

Table 3 summarizes the values of compressive strength of adobes as a function of rice husk content. It should be noted that the initial tangent Young’s modulus E is also calculated as the slope of the curve between 0.2 and 0.8 $f_{c}$ [25].

**Figure 13:**
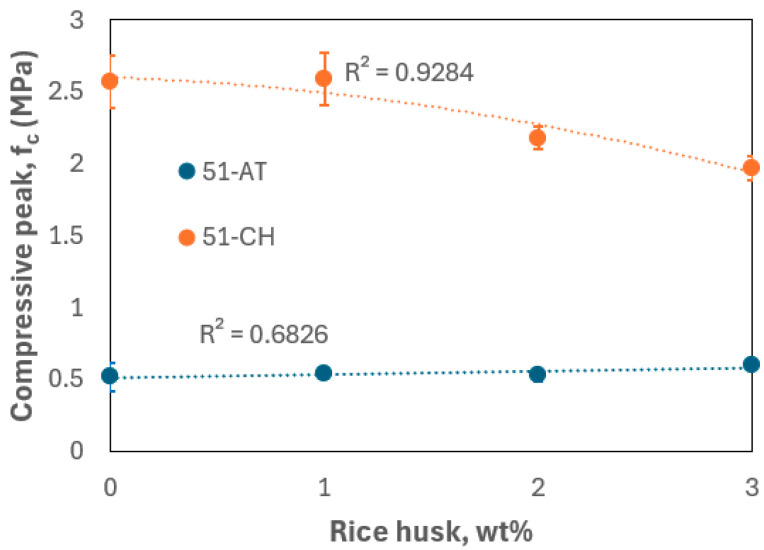
Compressive strength vs. rice husk content.

Figure 13 illustrates the evolution of peak compressive strength as a function of rice husk content. The two raw soils display markedly different compressive strengths (2.57 MPa for 51-CH and 0.52 MPa for 51-AT), which, as previously noted, are attributed to differences in their mineralogical compositions. For 51-CH, little variation is observed between 0 and 1 wt%, followed by a gradual decline in peak strength up to 3 wt%, reaching 1.97 MPa. In contrast, for 51-AT, the peak compressive strength increases slightly with rice husk content, reaching 0.6 MPa at 3 wt%. Nevertheless, all recorded values exceed the minimum threshold of 0.6 MPa recommended for adobe bricks used in earthen construction [32], as well as the 0.3 MPa minimum specified for earthen masonry walls [46].

For the calcareous soil 51-AT, it is suggested that the slight improvement in compressive strength with the addition of rice husk—peaking at 3 wt%—is due to the structural role played by the fibers. Compared to the siliceous soil 51-CH, 51-AT is mechanically weaker and less cohesive because of its high carbonate content. In this context, the rice husk fibers contribute positively by limiting crack propagation and enhancing the mechanical integrity of the granular skeleton. They reinforce internal cohesion in a matrix that lacks inherent binding capacity. Although porosity also increases (up to 37.97%), it does not undermine the strength of the material; instead, it allows for better anchoring of the fibers, supporting improved fiber–soil interaction and compensating for the weak cohesion of the original soil.

For both types of raw earth, compressive strength decreases at a rice husk content of 2 wt%. Similar findings were reported by Samson et al. [15] and Sutas et al. [21], who concluded that 1 wt% of rice husk was the optimal content for enhancing the compressive strength of adobe bricks. In a study on the performance of earth blocks reinforced with barley straw, Bouhicha et al. [53] clearly observed that reinforcement up to approximately 1.5 wt% improved compressive strength by 10 to 20%, depending on the soil’s characteristics (plasticity index, elemental composition, and particle size distribution). However, further increases in fiber content appeared to reduce the strength. Other researchers have even reported that compressive strength peaks at fiber or aggregate contents below 1 wt% [14,16]. In the literature review by Laborel-Préneron et al. [54] on the use of plant aggregates and fibers in earthen construction, it is highlighted that the content and behavior of these materials within the soil matrix are highly variable. Some authors reported that compressive strength decreased with increasing material porosity [55], while others attributed lower compressive strength to the use of certain aggregates or fibers due to poor adhesion between the particles and the clay matrix [56,57]. Indeed, the key factor influencing the mechanical performance of these composites is the fiber–matrix adhesion, as reported by Segetin et al. [58]. Even when the individual constituents possess favorable properties, effective fiber/matrix bonding is essential for proper load transfer within the composite [59]. According to Ouedraogo et al. [14], the strong adhesion of rice husks to the clay matrix is facilitated by their rough surface texture and high silica content. In fact, the compressive strength at 1 wt% rice husk for the clay-rich soil from Châlons is significantly higher than values reported in studies on adobes reinforced with kenaf fibers [60] or fonio straw [61]. Overall, our results are consistent with these findings in the literature.

**Figure 14:**
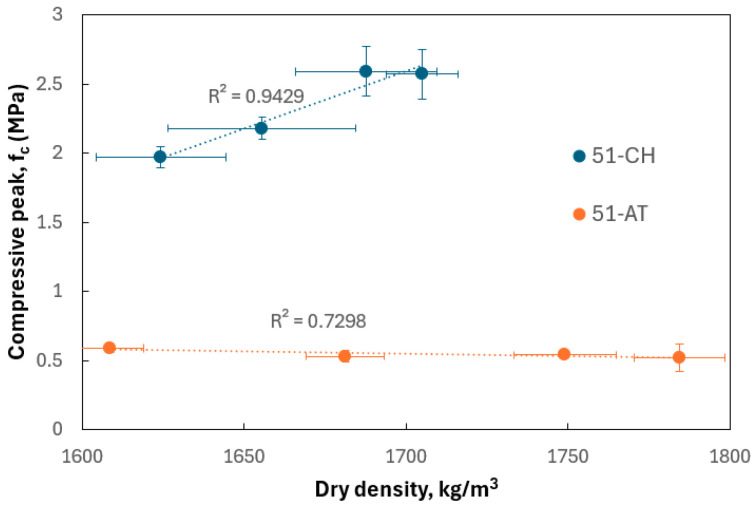
Compressive strength vs. dry density.

The previous results can also be inversely corroborated by examining the dry density, as shown in Figure 14. The addition of rice husk, which contributes to the material’s lightening, exerts a differing influence depending on the soil type. For 51-AT, only a marginal variation in peak compressive strength is observed as a function of density. In contrast, for 51-CH, the peak strength increases logically with rising density, reflecting the direct relationship between density and mechanical performance in this more cohesive, less carbonate-rich soil.

Furthermore, Table 3 shows that the tangent Young’s modulus followed the same trend as the compressive strength. For sample 51-CH, the modulus decreased with increasing rice husk content, with the highest value recorded at 151.83 MPa for 1 wt% rice husk. In contrast, for sample 51-AT, the maximum Young’s modulus (27.99 MPa) was achieved at 2 wt% rice husk. During compression testing, the 51-AT specimens exhibited more cracking compared to 51-CH. This may be attributed to the fact that, in the 51-CH composite, rice husks form bridges with the clay matrix and are more compressible, thereby generating a higher residual strength. This phenomenon was observed during specimen failure, where loss of adhesion between the two materials was evidenced by the detachment of rice husks [54].

### 3.6. Thermal and Hydric Analyses

The results for thermal conductivity (λ), thermal diffusivity (a), and specific heat capacity (C_p_) are presented in Table 4. Thermal conductivity (λ) quantifies a material’s insulating capacity—the lower the conductivity, the greater the material’s ability to insulate. Thermal diffusivity (a) represents a material’s ability to transmit temperature variations between two surfaces; a lower diffusivity indicates slower heat transfer through the material. Specific heat capacity (C_p_) characterizes the material’s ability to store thermal energy—higher values correspond to greater heat storage capacity [59].

The thermal conductivity of insulation materials derived from natural fibers generally ranges between 0.03 and 0.06 W/(m·K) [62]. Rice husk is peculiar in that it is composed of a mixture of organic matter such as cellulose and lignin with high levels of insulation capabilities, and mineral components, mainly including amorphous silica, on the order of 20% [63], which explains why rice husk displays a thermal conductivity of 0.066 W/(m·K). In contrast, earth—though superior to other conventional masonry materials—exhibits limited insulation capacity, with thermal conductivity values of 0.782 W/(m·K) and 0.879 W/(m·K) for 51-AT and 51-CH, respectively.

**Figure 15:**
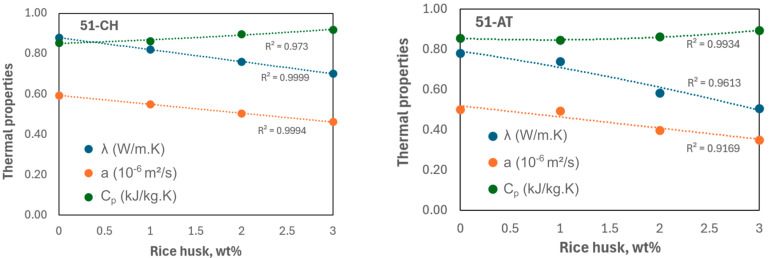
Thermal characteristics vs. rice husk content for 51-CH (**left**) and 51-AT (**right**).

To evaluate the thermal impact of incorporating a bio-based insulating material into earth, Figure 15 presents the evolution of the thermal parameters of the earth–rice husk composite as a function of rice husk content, up to 3 wt%.

Thermal testing results demonstrated that both the thermal conductivity (λ) and thermal diffusivity (a) of the adobes decrease monotonically with increasing rice husk content, whereas the specific heat capacity (C_p_) increases as the proportion of rice husk rises in the mixture. At a concentration of 3 wt% rice husk, the thermal conductivity reached 0.508 W/(m·K) for 51-AT and 0.701 W/(m·K) for 51-CH, representing reductions of 35% and 20%, respectively, compared to the raw earth material. These outcomes reveal a substantial enhancement in the insulating performance of the adobes due to the incorporation of rice husk, as already shown by Antunes et al. [17]. This improvement in thermal conductivity can be attributed to the progressive reduction in dry density with increasing rice husk content, leading to greater porosity in the composite material, as shown in Table 2. This relationship between increased porosity and reduced thermal conductivity has also been confirmed in the literature for other bio-based composite formulations [54,64,65,66,67]. Despite being reduced, thermal conductivity remains too high to consider adobes lightened with rice husk as excellent insulating materials, the threshold of which is at 0.03 W/(m·K) by standard DIN 4108 [68]. Should this type of adobe be used as load-bearing construction material in walls, it would be necessary to further improve the insulation properties at the wall scale by creating additional insulation layers [51].

Raw earth is also valued for its high thermal inertia, which refers to its ability to delay heat transfer. Therefore, a lower thermal diffusivity indicates better resistance to temperature fluctuations. Rice husk possesses inherently better thermal properties than raw earth, with a measured diffusivity of 3.54 × 10^−7^ m^2^/s, compared to 5.03 × 10^−7^ m^2^/s for soil sample 51-AT and 5.93 × 10^−7^ m^2^/s for 51-CH. When 3 wt% rice husk is added to the raw earth, the resulting composite exhibits thermal diffusivities of 3.51 × 10^−7^ m^2^/s for 51-AT and 4.62 × 10^−7^ m^2^/s for 51-CH, corresponding to reductions of 30% and 22%, respectively. For comparison, commonly used masonry materials such as solid bricks and concrete exhibit higher diffusivities of 6.1 × 10^−7^ m^2^/s and 8.3 × 10^−7^ m^2^/s, respectively. Consequently, it can be stated that bio-based earthen materials enhance the thermal comfort of the inhabitants.

Finally, the specific heat capacity of a material corresponds to the amount of energy required to raise the temperature of a unit mass by one degree Celsius. With the addition of 3 wt% rice husk, the specific heat capacity increased by 9.81% for adobe 51-AT and by 7.74% for 51-CH. These values are consistent with findings reported in previous studies [48,69,70]. In agreement with these studies, it can be inferred that the more insulating a material is, the more heat it is capable of absorbing. Accordingly, the specific heat capacity of the adobes increases as their density decreases, since plant fibers possess a higher heat capacity than mineral components [64].

**Figure 16:**
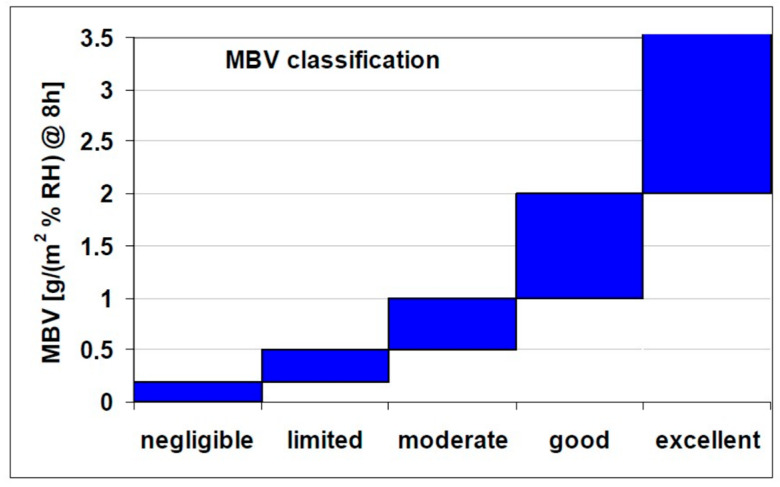
Classification of moisture buffer values (from Rode [33]).

Regarding the hydric properties of the studied samples, reference is made to the measurement of the moisture buffering value (MBV), which reflects a material’s ability to regulate indoor humidity. This capability contributes to improved air quality, reduces microbial growth, and subsequently lowers potential risks to respiratory health. The Nordtest project [33] established a classification system for moisture buffering values, ranging from negligible to excellent, to assess a material’s capacity to moderate indoor humidity. This classification is illustrated in Figure 16.

**Figure 17:**
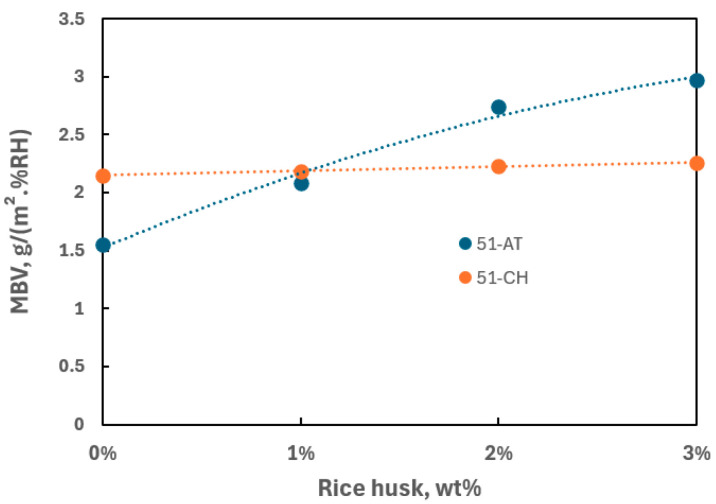
Moisture buffer value of biocomposites vs. rice husk content.

The values of MBV deduced from Equation (1) for all samples are summarized in Table 2 and represented as a function of rice husk content in Figure 17.

Raw calcareous 51-AT soil can be considered a good moisture regulator with MBV = 1.55 g/(m^2^·%RH), while raw 51-CH soil appears as an excellent regulator with MBV = 2.15 g/(m^2^·%RH). Furthermore, McGregor et al. [71] stipulated that the moisture performance could potentially be improved with the addition of organic aggregates. In the present study, regardless of the soil origin, an increase is observed in the moisture performance as rice husk is added to the mixture. The observations made on the 51-AT composite reveal a strong increase in MBV (+91.6%), up to 2.97 g/(m^2^·%RH) for 3 wt% rice husk. Conversely, the MBV of siliceous 51-CH samples slightly increases (+5.1%) as rice husk content increases. Adding rice husk categorizes both composites as excellent moisture regulators. Whether strongly or slightly significant, the performance level can be assumed to be related to the elemental composition of raw soils, a siliceous soil yielding strong benefits, while a calcareous one results in modest benefits. This last comment remains to be confirmed over a broader range of soil compositions.

### 3.7. Acoustic Tests

**Figure 18:**
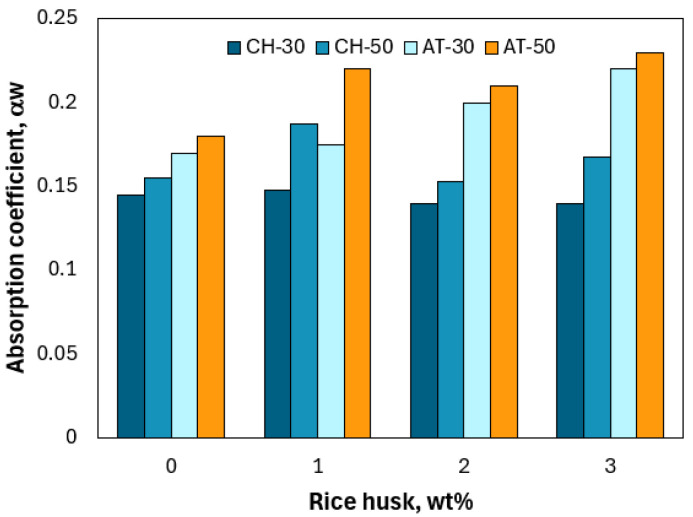
Weighted acoustic absorption coefficient $\alpha_{w}$ vs. rice husk content.

Figure 18 presents the evolution of the weighted sound absorption coefficient $\alpha_{w}$ for all adobe samples as a function of the rice husk content (wt%) in the mixture. Two different heights of cylindrical specimens, namely, 30 and 50 mm, were investigated. The calculation of the coefficient $\alpha_{w}$ is based on ISO 11654:1997 [72], which allows the conversion of the frequency-dependent acoustic absorption data into a single evaluation index. Annex B of the same standard provides an acoustic classification of materials according to the $\alpha_{w}$ values, ranging from “extremely absorbing” for values close to 1.0 to “reflecting” for values approaching 0.

The acoustic absorption coefficient varies with both the specimen thickness and the rice husk content in the mixture (Figure 18). Overall, the addition of rice husk does not significantly alter the acoustic performance of the biocomposite, which remains within a very low classification E, ranging from “hardly absorbing” to “reflecting.” Indeed, for all the tests conducted, the weighted absorption coefficient was calculated to fall between 0.14 and 0.23. This result can be attributed to the condition of the surface directly exposed to sound waves: since the samples were smoothed during fabrication, they lack surface porosity and specific roughness that would otherwise contribute to sound attenuation. McGrory et al. [73] stated that a correlation exists between the thickness of the samples and the absorption coefficient values, which is also observed in both biocomposites investigated herein. However, this trend seems to only be observed for samples with limited thickness, as reported by Badouard [74], who observed that $\alpha_{w}$ no longer evolved beyond a thickness of 80 mm for samples of biocomposite formulated from grape pressing by-products.

In comparison with other commercial insulation materials, Badouard reported weighted absorption coefficient values of 0.6 for rock and glass wool (thickness: 30 mm) and 0.35 for polyurethane foam (thickness: 35 mm). It can be concluded that adobe lightened by the addition of rice husk does not offer any advantage in terms of sound absorption capabilities, with levels comparable to those of smoothed concrete.

### 3.8. Comparison of Rice Husk and Other Natural Fibers

Table 5 summarizes the influence of rice husk content on the Multiphysics properties of adobes. Green cells denote a beneficial gain on the performance considered, while red cells characterize a detrimental benefit.

It appears that using rice husk addition in order to lighten the material mostly affects the hygrothermal properties of the composite, namely, the thermal conductivity and moisture buffering value. Mechanically speaking, the benefits are globally modest or even detrimental, depending on the raw soil used. In terms of acoustic insulation, no significant effect is observed, regardless of the ratio of natural component used.

Many different vegetal fibers can be used to improve the performance of adobes. Outcomes may greatly vary depending on the type of soil and the fiber content. Table 6 summarizes data from the literature and compares the properties of various soil-based biocomposites.

### 3.9. Comments on Agricultural Waste Management

The world’s four most widely cultivated crops—sugarcane, maize, cereals, and rice—generate substantial quantities of agricultural waste, the management of which presents global challenges. Rice husk may be marginally used in its unburnt current state (gardening mulch, animal bedding) but is mainly randomly burnt for crop drying, energy production, and biochar [80]. In regions with high rice production, the open burning of rice residues remains prevalent, significantly contributing to air pollution and posing serious risks to both human health and the environment. This practice leads to the release of greenhouse gases (GHGs), notably carbon dioxide (CO_2_), nitrous oxide (N_2_O), and methane (CH_4_).

To assess the environmental relevance of incorporating rice husk into construction materials (excluding insulation applications), it is useful to estimate the potential volumes involved. In France, annual rice husk production is estimated at 65,000 tons [81], corresponding to approximately 6.5 m^3^ per hectare. In the present study, up to 3 wt% of rice husk was incorporated into the raw earth adobe formulations, which equates to 27.1 vol% for 51-AT and 26.3 vol% for 51-CH, calculated using the following equation:

(2) $$vol\%=\frac{100}{1+\left(\frac{100}{wt\%}-1\right)\frac{\rho_{h}}{\rho_{soil}}}$$

where $\rho_{h}$ and $\rho_{soil}$ denote the bulk densities of rice husk and soil, respectively.

Consider a single-story house of 120 m^2^ with 3 m high walls and a wall thickness of 30 cm constructed from earth adobes lightened with rice husk. A rough estimate, based on the aforementioned volumetric fractions, suggests that approximately 21 m^3^ of rice husk would be required, corresponding to about 3.171 tons. Given that the calcination yield of rice husk is approximately 20% and that each kilogram of rice husk ash generates 1.72 kg of CO_2_ equivalent [82], it can be inferred that, for the aforementioned dwelling, incorporating rice husk into earthen construction materials rather than open-air incineration would prevent the emission of approximately 1090 kg of CO_2_eq into the atmosphere. Extrapolated to a larger scale, utilizing rice husk as a construction material rather than burning it, which remains the predominant practice in major rice-producing countries, could result in a significantly reduced environmental footprint.

## 4. Conclusions

This study explored the potential of rice husk as a bio-based additive to enhance adobe bricks, focusing on mechanical, hygrothermal, and acoustic properties, using 1, 2, and 3 wt% rice husk in two soil types—siliceous (51-CH) and calcareous (51-AT).

Mechanically, rice husk had soil-dependent effects. In 51-CH, compressive strength peaked at 1 wt%, suggesting improved cohesion and fiber–matrix interaction. This hypothesis requires confirmation through microscope observations on finely surfaced biocomposite samples, a perspective for future studies. For 51-AT, strength was lower but slightly improved at 3 wt%, likely due to enhanced ductility and porosity. However, all formulations met strength thresholds for earthen construction.

Thermal results showed that increasing rice husk content reduced thermal conductivity and diffusivity while increasing heat capacity, improving insulation and thermal inertia. At 3 wt%, conductivity dropped by up to 35% for 51-AT and 20% for 51-CH, demonstrating clear thermal benefits.

Moisture buffering was already good to excellent in raw soils and likely improved further with rice husk due to increased surface porosity. Acoustic performance remained low across all samples, limited by smooth surfaces and low macro-porosity.

Environmentally, the reuse of rice husk helps avoid open-air burning, cutting CO_2_ emissions and promoting circular, low-impact building methods.

In summary, rice husk shows promise as a stabilizer for adobe bricks, particularly for enhancing hygrothermal performance, with optimal dosage depending on soil characteristics and performance goals. Future work will explore the incorporation of rice husk ash—rich in silica—to further improve mechanical properties. A larger-scale study is planned, potentially including simulation, wall-scale characterization, and in situ deformation measurement using digital image correlation.

## Figures and Tables

**Table 1:** Elemental composition of soils and rice husk (Atomic %).

	O	Ca	Si	Al	Fe	K	P	Mg	Si/Ca
51-AT	71.9	21.5	3.2	0.7	0.3	0.26	-	0.32	0.15
51-CH	70.16	9.17	15.77	3.25	0.93	0.8	-	0.43	1.72
Rice husk	74.8	0.27	24.25	0.03	0.02	0.47	0.1	0.1	89.81

**Table 2:** Physico-chemical properties of soils and rice husk.

	Rice Husk	51-CH				51-AT			
		0 wt%	1 wt%	2 wt%	3 wt%	0 wt%	1 wt%	2 wt%	3 wt%
Apparent density (kg/m^3^)	150.9	1736.87	1714.65	1680.06	1651.43	1814.73	1763.61	1693.02	1618.90
Dry density (kg/m^3^)	137.6	1704.91	1687.73	1655.53	1624.19	1784.42	1748.97	1681.24	1608.48
Absolute density (kg/m^3^)	1284	2720.17	2705.81	2691.45	2677.09	2651.00	2637.33	2623.66	2609.99
Moisture content (%)	8.27	1.84	1.57	1.46	1.65	1.67	0.83	0.70	0.64
Porosity (%)	88.32	36.15	36.63	37.58	38.31	31.55	33.13	35.47	37.97
Clay content (%)	-	11.3	-	-	-	10.27	-	-	-
pH	6.87	7.9	-	-	-	7.7	-	-	-
CaCO_3_ (%)	-	41.60	-	-	-	83.62	-	-	-
Organic matter (%)	-	0.24	-	-	-	1.50	-	-	-
MBV g/(m^2^·%RH)	-	2.15	2.18	2.23	2.26	1.55	2.09	2.74	2.97

**Table 3:** Mechanical performance of studied soils.

	51-CH				51-AT			
	0 wt%	1 wt%	2 wt%	3 wt%	0 wt%	1 wt%	2 wt%	3 wt%
$\mathrm{Peak}\;\mathrm{stress}\;f_{c}$ (MPa)	2.57 (0.18)	2.59 (0.18)	2.18 (0.08)	1.97 (0.08)	0.52 (0.10)	0.54 (0.02)	0.53 (0.04)	0.60 (0.02)
$\mathrm{Peak}\;\mathrm{strain}\;\varepsilon_{u}$ (%)	2.32 (0.79)	2.39 (0.60)	3.18 (0.76)	2.84 (0.67)	2.76 (0.06)	2.92 (0.33)	2.39 (0.49)	3.29 (1.16)
Mean tangent modulus E (MPa)	146.38 (44.63)	151.83 (41.85)	98.58 (17.68)	95.81 (26.08)	20.60 (5.21)	19.70 (2.84)	27.99 (6.18)	27.02 (12.06)

**Table 4:** Thermal characteristics of soils and rice husk.

	Rice Husk	51-AT				51-CH			
		0 wt%	1 wt%	2 wt%	3 wt%	0 wt%	1 wt%	2 wt%	3 wt%
Thermal conductivity λ (W/m·K)	0.066 (0.001)	0.782 (0.021)	0.740 (0.045)	0.583 (0.042)	0.508 (0.034)	0.879 (0.029)	0.821 (0.015)	0.760 (0.026)	0.701 (0.022)
Diffusivity a (10^−6^ m^2^/s)	0.354 (0.009)	0.503 (0.030)	0.496 (0.020)	0.399 (0.029)	0.351 (0.023)	0.593 (0.031)	0.550 (0.008)	0.503 (0.014)	0.462 (0.010)
Specific heat capacity $C_{p}$ (kJ/kg·K)	1.234 (0.057)	0.856 (0.033)	0.846 (0.020)	0.863 (0.014)	0.94 (0.004)	0.853 (0.015)	0.863 (0.011)	0.898 (0.009)	0.919 (0.012)

**Table 5:** Synthesis of the benefits brought by rice husk addition (green stands for a beneficial effect, red for a detrimental effect and white denotes an invariant effect).

	Compressive Peak			Thermal Conductivity			Acoustic Absorption			Moisture Buffering Value		
RH, wt%	1%	2%	3%	1%	2%	3%	1%	2%	3%	1%	2%	3%
51-AT	↗	↗	↗	↘	↘	↘	→	→	→	↗	↗	↗
51-CH	↗	↘	↘	↘	↘	↘	→	→	→	→	→	→

**Table 6:** Characteristics of rice husk compared to other vegetal fibers in raw earth composites.

Fiber Used	wt%	Dry Density (kg/m^3^)	Compressive Strength (MPa)	Thermal Conductivity (W/m·K)	MBV (kg/(m^2^·%RH))	Weighted Sound Absorption Coefficient	References
Reference (raw soil)	0	1705–1784	0.52–2.57	0.782–0.879	1.5–2.2	0.14–0.18	Present study
Rice husk	0.2–1	1770–1900	2.4–3.65	0.8–1.1	-	-	[14]
	0.75	-	3.93	-	-	-	[16]
Rice husk studied	1–3	1608–1749	0.52–2.59	0.51–0.821	2.09–2.97	0.16–0.23	Present study
Wheat straw	0.72–3.84	1430–1830	0.5–1.5	-	-	-	[75]
	1–4	1393–1701	3.08–4.93	0.294–0.411	-	-	[76]
Fonio straw	0.2–1	-	2.3–2.8	0.35–1.1	-	-	[61]
Coconut fiber	0.25–1	1795–1857	1.4–2.7	-	-	-	[4]
	1–2	1675–1715	1.95–2.05	-	-	-	[77]
Sugarcane bagasse	0.25–1	1808–1867	1.1–2.05	-	-	-	[4]
Harakeke (flax)	0.6–0.8	-	2.0–3.5	-	-	-	[58]
Date palm	1–5	1250–1679	2.1–3.6	0.316–0.514	-	-	[78]
Bunho and junco	1–3	1278–1445	0.355–1.617	0.30–0.55	-	-	[79]

## Data Availability

The original contributions presented in this study are included in the article. Further inquiries can be directed to the corresponding author.
